# Editorial: Magnetic neurophysiology: the cutting edge of real time neurodiagnostic technology

**DOI:** 10.3389/fmedt.2025.1682837

**Published:** 2025-10-06

**Authors:** Pegah Afra, Shigenori Kawabata, Glenn D. R. Watson, Timothy P. L. Roberts

**Affiliations:** ^1^ Department of Neurology, UMass Chan Medical School, Worcester, MA, United States; ^2^ Department of Advanced Technology in Medicine, Institute of Science Tokyo, Tokyo, Japan; ^3^ Department of Orthopaedics and Spine Surgery, Institute of Science Tokyo, Tokyo, Japan; ^4^ Department of Psychology and Neuroscience, Duke University, Durham, NC, United States; ^5^ Department of Radiology, Program in Advanced Imaging Research and Lurie Family Foundations MEG Imaging Center, Children’s Hospital of Philadelphia, Philadelphia, PA, United States; ^6^ Department of Radiology, Perelman School of Medicine, University of Pennsylvania, Philadelphia, PA, United States

**Keywords:** magnetosensors, superconducting quantum inteference devices (SQUIDs), opticlaly pumped magnetometers (OPMs), magnetoencephalagraphy (MEG), magnetospinography (MSG), magnetoneurography (MNG)

**Editorial on the Research Topic**
Magnetic neurophysiology: the cutting edge of real time neurodiagnostic technology

Magnetoneurophysiology, the study of magnetic fields generated by neural activity, has experienced a significant evolution in recent years due to advances in sensor technology and a renewed focus on clinical applications. The field is rooted in fundamental biophysics, namely the intra-axonal currents of action potentials that produce measurable magnetic fields. While these principles have long been understood, emerging technologies such as optically pumped magnetometers (OPMs) and improved superconducting quantum interference devices (SQUIDs) have transformed both the precision and accessibility of magnetographic recordings.

This editorial brings together a series of reviews and research contributions on our research topic that collectively chart the trajectory from foundational principles to cutting-edge innovations in magnetoencephalography (MEG), magnetospinography (MSG), magnetoneurography (MNG), and their combination. These developments not only enhance technical performance but also open the door to novel, more naturalistic paradigms for studying brain, spinal cord, and peripheral nerve activity, which can shape the future of clinical neurophysiology.

## 1 Basic magnetoneurophysiology

The intra-axonal flow of the propagating action potential generates a magnetic field that is unaffected by the volume-conducting properties of the surrounding tissue. However, signal decay with distance remains an issue (1). A review by Adachi and Kawabata provided a clear explanation of the fundamentals of magnetoneurophysiology. Specifically, they highlighted the mechanisms that underlie magnetic fields traveling through the spinal cord and peripheral nerves. These include transmembrane inward and outward currents generated during action potentials, along with intracellular leading and trailing currents and extracellular volume currents. These relationships are clearly described and illustrated in Figure 2 of their article. Although these concepts have been extensively studied in clinical neurophysiology, they have rarely been depicted in a single, easy-to-comprehend figure.

## 2 Magnetic sensor technology

The different sensor-level technologies are discussed in detail in our research topic. Adachi and Kawabata began with the fundamentals of the inaugural SQUID sensors. They depicted the ring-shaped superconducting structure and the weak Josephson Junction. They explained SQUID magnetometry and how the DC SQUID magnetometers indirectly measure magnetic fields. The biophysical signals constitute the external magnetic flux and penetrate the ring, generating a shielding current in the opposite direction. This results in the generation of a superconducting critical current and voltage across the Josephson junctions, enabling the measurement of magnetic flux by the SQUID sensors. In the article, they explained the requirement for cryogenic thermal insulation to achieve superconductivity and, therefore, keep the sensors operational. Thereafter, the authors reviewed the latest version of SQUID sensor technology: vector-type SQUID magnetometers, consisting of three orthogonally oriented pickup coils coupled with discrete SQUIDs (one axial and two planar). This design allows for the assessment of magnetic fields normal to the body surface along with tangential components (Adachi and Kawabata).

Bonnet et al. discussed OPM-based sensors and compared them to SQUID-based sensors. Optically pumped magnetometers measure the transmission of monochromatic light (lasers) through a glass cell container (called a cell) of atoms with finite spin angular momentum and zero orbital angular momentum (2). A cell contains either vapor of a sensitive element [such as an alkali metal like ^87^Rubidium (^87^Rb)], which must be heated to the vapor phase, or ^4^He gas (Bonnet et al., 2), which does not require heating. The light is circularly polarized in alkali-OPMs (e.g., Rb-OPMs) and linearly polarized in He-OPMs. To achieve the Spin Exchange Relaxation Free (SERF) operating mode of alkali (^87^Rb) OPMs, the cell filled with alkali gas has to be heated to 150 °C, while the He-OPMs function at room temperature (2, 3). Overall, the bandwidth of Rb-OPMs is DC-200 Hz and the bandwidth of He-OPMs is DC-2KHz. The dynamic range of Rb-OPMs is ∼5–15 nT while in open-loop mode of operation and can be ∼150 nT in closed-loop mode (Bonnet et al., Roberts et al., 3, 4). The dynamic range of He-OPMs is ∼200 nT. Differences in thermal insulation requirements, the nature of the vapor cell, open-loop vs. closed-loop operation and electronics design and implementation lead to significant differences between OPM sensors even those of the same vapor type. Moreover, fundamental considerations such as “noise level” differ between systems, with, for example, He-OPMs typically characterized by noise levels two to three times greater than those of Rb counterparts. Thus, key parameters have to be assessed critically when designing OPM systems for MEG. These include differences in operating **bandwidth** (for the range of signals that MEG can detect—typical clinical applications consider activity in the 0.1–100 Hz range), and signal-to-noise ratio (dependent on the **noise level**, which is typically <10 fT/√Hz for conventional SQUID systems, approaching this level for Rb-OPMs (∼10–15 fT/√Hz), but 2–3× more challenging for He-OPMs, ∼30–50 fT/√Hz) (Bonnet et al., Roberts et al., 2–5). Furthermore, the **dynamic range** of the detectors, and their associated electronics, influences their tolerance of different ambient magnetic fields (and thus the degree to which the arrays can “move”, with the participant, through spatially varying fields). These differences have implications for clinical applications: SQUID sensors require a rigid helmet in which the sensors rest in a cryogenic liquid-He dewar (resulting in increased distance of sensors from the source, with concomitant loss of sensitivity). On the other hand, heated Rb-based OPMs require thermal insulation and/or airflow, while the He-based systems, do not have any reason for distancing and can be placed close to the scalp. In clinical practice, with the thin insulation, Rb-OPMs can also be considered “scalp-mounted”. The main advantage of SQUID sensors is their bandwidth (DC-up to 40 kHz) compared to Rb-OPMs (DC-200 Hz) and He-OPMs (DC-2 kHz), although given the range of signals detected from the human brain, this advantage may currently be considered of little practical impact. Optically pumped magnetometer detector systems are also characterized by a wide variation in dynamic operating range from ∼5 nT to up to 300 nT, often dependent on their operation in closed loop (vs. open loop, with a lower dynamic range); however, other factors such as feedback electronics influence the achievable dynamic range. While the lower limit of this may preclude movement through spatially varying magnetic fields, Roberts et al. demonstrated that some commercial systems indeed have sufficient dynamic range (±150 nT) to tolerate head movement and allow effective recording from a moving head. Nonetheless, similar to SQUIDs, OPM sensors currently have to operate in a magnetically shielded room (MSR), in which spatial variations in the magnetic field can typically be maintained at less than 100nT. Thus, the OPM array (and the participant) can move to different positions in the room (provided the OPM sensors have an appropriate dynamic range >∼100 nT). Spedden et al. discussed cross axis projection errors (CAPEs) and how the large background magnetic noise can introduce gain and orientation errors in Rb-based OPM output signals for some systems that require field nulling systems (closed loop mode of operation, where internal coils null the field), although technical differences do exist even within different Rb-OPM systems, with some systems demonstrating robustness to these concerns. Bonnet et al. explained that He-based OPMs due to their large dynamic range result in less saturation and therefore require no additional field nulling systems, although as shown by Roberts et al., Rb-OPM systems with sufficient dynamic range can also operate effectively without additional external field nulling.

## 3 Magnetography technology

In the cryo-space, Roberts et al. reviewed traditional SQUID-based MEG systems, including the requirement for a rigid helmet to host SQUID-based sensors in a cryo-bath. Practical constraints include the need to maintain vacuum and thermal insulation within the dewar, which translates to the physical inflexibility of the fixed sensory array, and the need for dedicated infant, child and adult-sized MEG dewars, each adding to the cost of an MEG laboratory, in addition to the cost of maintaining vacuum and the weekly helium refills required to maintain the cryo-bath.

Adachi and Kawabata discussed the latest SQUID-based MSG/MNG system, which is suitable for recording magnetic fields from the spinal cord, plexuses and peripheral nerves. This system employs a new kind of machine that has never been available before. The cryogenic MSG/MNG developed by the Kanazawa Institute of Technology (KIT) in collaboration with the Tokyo Medical and Dental University (TMDU, now the Institute of Science Tokyo) has undergone several iterations. It is the only SQUID MSG/MNG machine that is ready for clinical use. It requires a magnetically shielded room and allows for frontal and lateral x-ray imaging while maintaining the same posture for measurements. The elegantly engineered sensor array is mounted along the upper surface of the cryostat, which hosts 44 sensor locations and, with 3-axis detectors, has 132 channels that are curved to originally conform to the dorsal surface of the neck. This evolution of the cryostat structure allows for a closer proximity of SQUID sensors to the cervical/lumbar spine and the extremities of different-sized subjects (Adachi and Kawabata)

Spedden et al. introduced the OP-MSEG (i.e., magnetospinoencephalography) system, the first machine to image the brain and spinal cord while simultaneously collecting EMG. The system can use generic or custom-built 3D printed scanner casts that house Quspin-manufactured triaxial OPMs (Louisville, CO) in custom arrays for the head and back; the recordings are performed in shielded rooms. Bonnet et al. discussed a He-OPM-based MEG system with triaxial sensors in up to 97 positions. The head cap is flexible with a tightening system to adjust the sensor array so that it fits all head shapes and sizes. There are up to 97 positions for adult head sizes and 89 positions for child head sizes.

## 4 Clinical applications

Our research topic series covers the most recent advances in the clinical applications of cryogenic and optically pumped magnetometry machines:

Ghahremani et al. reviewed the fundamentals of motion artifacts in biomagnetic measurements. The authors discussed the complexity of these motion artifacts and how environmental conditions, including the ambient magnetic field, influence their magnitude, and therefore the recording rooms shielding factor. They also evaluated the variability of these artifacts and the effectiveness of gradiometers in reducing them.

Adachi and Kawabata discussed the only Cryogenic SQUID MSG/MNG machine ready for clinical use in the spine and peripheral nerve spaces, providing neurophysiological access to locations that are less accessible by conventional neurophysiological recordings including the cervical, thoracic and lumbar spinal cords, the dorsal horn postsynaptic activity, the P9 component of the brachial plexus and the intra-tunnel segments of peripheral nerves.

Spedden et al. introduced the simultaneous recording of the cortex and spinal cord to study their natural rhythmic interactions. Proof-of-principle work was described by simultaneously recording from the spinal cord and brain after median nerve stimulation, in addition to longer latency spinal cord responses. The authors investigated the functional integration between the central nervous system and the periphery during simple movements.

Roberts et al. described the clinical advantages of OPM MEG systems beyond their improved sensitivity, including lack of need for cryogenic dewar resulting in scalp placement, being “wearable” and therefore allowing for “naturalistic” and “realistic” paradigms, allowing for movements beyond the artificial “button press” experiments, including simulated driving and target touch motor tasks and virtual reality stimulus paradigms. Roberts et al. described the potential clinical and research advantages of OPM MEG systems beyond their improved sensitivity. This includes lack of need for cryogenic dewar resulting in scalp placement and resulting in the sensor array being “wearable”. Since head motion can be tolerated (as the array maintains its relationship to the brain during movement), this allows for “naturalistic” and “realistic” paradigms, allowing for movements beyond the artificial “button press” experiments, including simulated driving and target touch motor tasks and virtual reality stimulus paradigms.

Bonnet et al. characterized the performance of He-OPM MEG systems in three head sizes (H50, H55, H58). The authors first simulated different sensor configurations with different numbers of channels in different head sizes and then assessed the signal-to-noise ratio and the source reconstruction accuracy, all using a phantom to simulate brain magnetic activity. They demonstrated that He-OPM MEG systems have equivalent detection capability and localization accuracy as SQUID-based MEG systems.

Taken together, these articles offer a technically principled overview of recent advances in magnetometry. Magnetospinography is emerging as a novel clinical application, initially implemented with a dedicated SQUID array, but further enabled by the flexible configuration offered by OPM sensor systems. While some technical differences may distinguish Rb-OPMs from He-OPMs (and, indeed, while technical differences in, for example, electronic systems can influence the performance of sensors of the same vapor type), both share opportunities to improve and expand the role of MEG enabled by scalp sensor placement, including enhanced sensitivity and motion tolerance. OPM-MEG systems thus offer the potential to study “traditional paradigms in non-traditional (moving) participants” (with promising applications to infants, young children and cognitively-impaired individuals, who were not collectively ideally-served by previous technologies), in addition to opening the experimental door to “naturalistic” paradigms, freeing them from the confines and constraints of traditional laboratory experimental designs. Ultimately, this contributes to a more robust connection between the brain and behavior.
